# Evolution of Cognitive Rehabilitation After Stroke From Traditional Techniques to Smart and Personalized Home-Based Information and Communication Technology Systems: Literature Review

**DOI:** 10.2196/rehab.8548

**Published:** 2018-03-26

**Authors:** José M Cogollor, Javier Rojo-Lacal, Joachim Hermsdörfer, Manuel Ferre, Maria Teresa Arredondo Waldmeyer, Christos Giachritsis, Alan Armstrong, Jose Manuel Breñosa Martinez, Doris Anabelle Bautista Loza, José María Sebastián

**Affiliations:** ^1^ Centre for Automation and Robotics UPM-CSIC Universidad Politécnica de Madrid Madrid Spain; ^2^ Life Supporting Technologies Universidad Politécnica de Madrid Madrid Spain; ^3^ Institute of Movement Science Department of Sport and Health Science Technische Universität München Munich Germany; ^4^ BMT Group Ltd London United Kingdom

**Keywords:** cognition, rehabilitation, stroke, eHealth, activities of daily living, delivery of health care

## Abstract

**Background:**

Neurological patients after stroke usually present cognitive deficits that cause dependencies in their daily living. These deficits mainly affect the performance of some of their daily activities. For that reason, stroke patients need long-term processes for their cognitive rehabilitation. Considering that classical techniques are focused on acting as guides and are dependent on help from therapists, significant efforts are being made to improve current methodologies and to use eHealth and Web-based architectures to implement information and communication technology (ICT) systems that achieve reliable, personalized, and home-based platforms to increase efficiency and level of attractiveness for patients and carers.

**Objective:**

The goal of this work was to provide an overview of the practices implemented for the assessment of stroke patients and cognitive rehabilitation. This study puts together traditional methods and the most recent personalized platforms based on ICT technologies and Internet of Things.

**Methods:**

A literature review has been distributed to a multidisciplinary team of researchers from engineering, psychology, and sport science fields. The systematic review has been focused on published scientific research, other European projects, and the most current innovative large-scale initiatives in the area. A total of 3469 results were retrieved from Web of Science, 284 studies from *Journal of Medical Internet Research*, and 15 European research projects from Community Research and Development Information Service from the last 15 years were reviewed for classification and selection regarding their relevance.

**Results:**

A total of 7 relevant studies on the screening of stroke patients have been presented with 6 additional methods for the analysis of kinematics and 9 studies on the execution of goal-oriented activities. Meanwhile, the classical methods to provide cognitive rehabilitation have been classified in the 5 main techniques implemented. Finally, the review has been finalized with the selection of 8 different ICT–based approaches found in scientific-technical studies, 9 European projects funded by the European Commission that offer eHealth architectures, and other large-scale activities such as smart houses and the initiative City4Age.

**Conclusions:**

Stroke is one of the main causes that most negatively affect countries in the socioeconomic aspect. The design of new ICT-based systems should provide 4 main features for an efficient and personalized cognitive rehabilitation: support in the execution of complex daily tasks, automatic error detection, home-based performance, and accessibility. Only 33% of the European projects presented fulfilled those requirements at the same time. For this reason, current and future large-scale initiatives focused on eHealth and smart environments should try to solve this situation by providing more complete and sophisticated platforms.

## Introduction

### General Framework

Imagine if people rejuvenated as time went by, such as Benjamin Button in the F Scott Fitzgerald's tale—a man who was an elder at birth and died with the appearance of a baby. Instead, human abilities deteriorate as they become older. Life expectancy at birth in Europe is increasing steadily [1], and by 2050, it is expected that 27% of the population will be older than 65 years [2]. Although this is a very positive outcome of the progress of medical care and the general improvement of our lives, it also imposes the great challenge of maintaining the overall (mainly cognitive) well-being of the aging population to sustain their functional capability, prolong their independent living, and reduce the risk of institutionalization.

Although aging involves several components, deterioration in cognitive abilities does not always appear due to aging, but rather, it often appears following a stroke. The risk of suffering a stroke duplicates in people aged above 65 years [3]. According to the statistical office of the European Union and the World Health Organization, the main causes of death in Europe in the last 15 years are heart attacks and strokes. Notably, in 2015, almost 6.24 million deaths were caused by stroke incidents [4].

Regarding those stroke survivors, as many as 68% of stroke patients meet the criteria for apraxia and action disorganization syndrome [5]. This deterioration usually makes people unable to remember their partial or full activities of daily living (ADL), and sometimes they even forget the complete execution of sequential actions. This means that this group of citizens is more dependent on caregivers and health care systems and they find it difficult to live independently [6]. Additionally, there are 2 other main syndromes derived from a stroke: hemiparesis and neglect.

With the objective of assisting the cognitive rehabilitation of stroke survivors, the traditional methodologies used to support the movements of the user act as a guide to be followed, and generally, they depend on the help of therapists. Currently, health care services are using centrally directed general guidelines which have very limited effect.

To address this issue at the cutting edge, some research and commercial home-based information and communication technology (ICT) systems (ie, MavHome, the Gloucester “Smart House,” ORCATECH, A2E2), mobile apps (ie, Garmin Connect, Endomondo, Newolo, Runtastic Results, Fitbit Trainer, Alfred), and Web-based strategies [7] have introduced more interactive approaches to health management and cognitive training by monitoring user activities and recommend actions.

The objective of this study was to provide an identification of the effective assessment of rehabilitation practices for cognitive disorders from a traditional perspective to a more technological one by presenting the most recent advanced eHealth systems and projects to not only maintain but also improve the cognitive status of both elderly people and stroke patients in their daily living.

Moreover, this study encompasses the concepts and manifestations in the execution of daily tasks of the main and most common stroke-related syndromes mentioned before, which can cause deficits in the execution of daily activities. They are presented in a broader context considering their influence in a high percentage of stroke survivors.

### Apraxia

It is thought that Hugo Liepmann presented the first description of apraxia as a distinct neuropsychological syndrome in the 19th century [8]. Liepmann’s theory showed deficient motor control at the heart of an apraxic impairment, and he defended the idea that some patients are not able to convert the image of an intended action into appropriate motor command even though they have the clear concept of what they want to do. Additionally, he considered apraxia as associated with left brain damage (LBD) after comparing studies with patient groups with left and right brain damages [8].

One of the most accepted definitions of apraxia is as follows: apraxia is a “disorder of skilled movement not caused by weakness, akinesia, differentiation, abnormal tone or posture, movement disorders (such as tremors or chorea), intellectual deterioration, poor comprehension, or uncooperativeness” [9]. This definition supports Liepmann’s idea of a disturbance at the interface between cognition and motor control, although some clinical manifestations make more emphasis in 3 main domains of human actions, namely, imitation of gestures, the performance of communicative gestures, and the use of objects.

The consequences of cognitive disorders in these domains have been studied mainly in specific activities which involve the use of tools and multistep tasks (see sections Action Disorganization Syndrome, Neglect, and Hemiparesis for details).

One of the main consequences of apraxia is the misuse of common everyday objects, for example, forks to eat soap, cutting paper with closed scissors, biting on the toothbrush when cleaning teeth, pressing knives into the loaf without moving it, or closing a paper punch on top of the sheet without inserting it. Although some apraxic patients present right-sided hemiplegia and their errors may be associated with the paralysis of the nondominant left hand, there is evidence of their pathological nature when observing the behavior of healthy people when manipulating objects with the nondominant hand [10,11].

The errors that appear when manipulating single tools, and those which are detectable in explicit testing, are probable to arise in the execution of multistep tasks also. However, there is a classification of specific errors associated with multistep tasks. The most relevant are as follows (see [11-14] for a more extensive classification):

- Misallocation: a correct action performed with the wrong recipient
- The omission of some steps
- Toying: the act of touching or briefly lifting objects not followed by goal-oriented manipulations
- Perplexity: hesitation before starting an action or failure when proceeding with an action

### Action Disorganization Syndrome

Action disorganization syndrome (ADS) is a neuropsychological disorder after a brain injury. Schwartz and colleagues were the first in providing a description [15]. The main characteristic of ADS is the high presence of cognitive errors when carrying out daily activities, such as preparing hot drinks, grooming, and dressing. However, these are not caused by a motor deficit [16]. On the basis of the study of Schwarz et al [15], ADS patients usually execute actions in the wrong sequence or select the wrong objects.

According to the studies presented in [15], 5 error types are considered to be common [17] and summarized in Table 1.

On the basis of different case studies with ADS patients, some errors are found to be more frequent in specific patients than others. In addition, thanks to these studies, it can be revealed that those patients who present high error rates commit more omission errors [18-20].

### Hemiparesis

It is found that 80% of stroke survivors suffer from hemiparesis. The main consequence of this disease is usually the weakness or inability to execute movements in one side of the patient's body. This inability can be centered in the user’s hands, facial muscles, arms, or even legs, leading to significant difficulty when carrying out their ADL [21].

When users present weaknesses in the body segments mentioned before, it is very probable that they will suffer from loss of balance due to muscle fatigue, coordination for walking, coordination when manipulating objects, and dexterity in achieving accuracy.

Obviously, the part of the body that experiences the inability depends on the part of the brain where the stroke happens. So, when an injury occurs on the left side of the brain, it usually results in deficits in right-sided components and vice versa.

### Neglect

Neglect is one of the consequences derived from a brain accident, typical of the left hemiplegic patient, that is, one who has suffered damage to the right hemisphere of the brain. It is often also recognized as unilateral spatial agnosia, hemi-inattention, or hemispatial neglect.

Neglect is a disorder of attention to space, especially with respect to space on the left. Neglect often appears as a problem exclusively visual in nature, because the difficulties of the left hemiplegic patient in orienting his or her eyes toward the visual field of the left are evident, especially during the first weeks following the stroke.

Neglect or hemi-inattention often represents an aspect that is not valued in terms of rehabilitation with due dedication but rather is defined as a phenomenon that in most cases resolves spontaneously after a few months following stroke.

However, neglect is not attributed to a problem in the ability to see but it is a problem related to attention. Already in 1874, Hughlings Jackson hypothesized that the right hemisphere was directly involved in perception and in relation to the outside world, and almost 100 years later, the neuropsychologist AR Lurija confirmed his perceptive peculiarities [22]. But even today, in contrast to the treatment of left hemiplegia, neglect is not taken into account because it is considered as an aspect that, in addition to being reduced spontaneously in most cases, is an element outside of the traditional motor re-education in hemiplegia.

### Studies About the Influences of Stroke in Activities of Daily Living

ADL comprises activities of basic self-care such as washing, grooming, and dressing, besides preparing drinks and food. The execution of these tasks involves sequences of more basic actions with manipulation of environmental objects directed at some desired end goal.

Table 2 shows a summary of some review of the literature on psychological studies of the production of sequential action in ADL tasks. They have generally been quite helpful to identify the nature of the errors committed by the patients with brain damage [15,19,23] or by healthy individuals, caused by different factors [24].

**Table 1:** Common error types based on studies by Cooper and Shallice [17].

Error	Explanation
Omission	Missing steps
Anticipation	Performance of actions in the wrong sequence
Quality errors	Action is carried out inappropriately
Object substitution	Misuse of objects
Place substitution	Movement of objects to wrong destinations

**Table 2:** Summary of previous studies of activities of daily living (ADL) tasks.

Institution and reference	ADL task	User
Philadelphia, United States [24]	Preparation of coffee	Neurologically healthy adults
University of Birmingham [16,25]	Preparation of tea, wrapping of a gift, preparation of a sandwich of cheese, and brushing teeth	Patients with action disorganization syndrome (ADS) and controls
University of Oxford [26]	Preparation of tea	Semantic dementia patient; ADS patient
University of Toronto, Canada [27]	Washing hands	Older adults with dementia
University of Nottingham [28]	Preparation of a hot drink	Stroke patients
University of London [29]	Preparation of coffee and tea	Neurologically healthy adults
Technical University of Munich [30,31]	Preparation of tea	Chronic stroke patients

Once these studies are analyzed, the main thought is that a strong correlation exists between those impairments in the execution of ADL and apraxia scores. The functional independence measure provides a rough evaluation of the performance of ADL at home or at hospitals, and this score was used to establish a correlation between outcome and apraxia [32]. A similar approach, the physical self-maintenance scale, is also used to measure the percentage of assistance by caregivers.

## Methods

### Areas Involved in the Review

Taking into account the behavior of stroke patients and with the purpose of providing a complete report on the evolution of methodologies for their screening and techniques of cognitive rehabilitation, a literature review was carried out by a multidisciplinary team of researchers from engineering, sports science, and psychology fields.

### Criteria

First of all, regarding the traditional methods for the individual evaluation of stroke patients as well as for providing cognitive rehabilitation after the screening, some material has been used from the results generated in some European projects that the authors collaborated in. In addition, a deep review of additional bibliography has been carried out. This additional review was designed using a systematic protocol to interpret and analyze the most relevant research [33,34]. For that purpose, 2 main questions were put on the table:

- Are the current methodologies for cognitive rehabilitation effective enough to improve health status and independence during the rehabilitation phase?
- Is the workload of the therapists in current rehabilitation techniques worthwhile based on the slow improvement of the cognitive abilities of patients?

Second, taking into account the negative answers to those questions, 2 additional ones were proposed:

- What types of technology are adequate and used nowadays to design a smart ICT–based system that would interact with stroke patients?
- How well could those technologies be received by end users, their families and carers, as well as health professionals?

Then, a new search of those actual ICT–based approaches or research projects that provide cognitive rehabilitation was done. For that purpose, using relevant keywords such as “cognitive rehabilitation,” “personalized health care,” and “stroke,” an extensive review of Web of Science and other sources (IEEE Xplore, *Journal of Medical Internet Research*) for research studies and Community Research and Development Information Service (CORDIS) [35] for research projects has been done. For example, 3469 results were retrieved from Web of Science focusing on cognitive rehabilitation after stroke (from 2012 onward), 284 results from *Journal of Medical Internet Research*, and 15 research projects from CORDIS were reviewed for classification and selection.

Finally, special attention was paid to the following features to classify the ICT–based approaches:

- Do they provide a home-based rehabilitation?
- Do they assist in the execution of complex ADL tasks?
- Do they present automatic error recognition?
- Are they accessible?

## Results

### Classification of Studies

Taking into account the review methodology described above, the material presented in this section focuses on (1) relevant classical techniques for the individual assessment of patients who suffer from cognitive disorders; (2) traditional methods to provide cognitive rehabilitation after screening; and, finally, (3) the most recent proof of concepts, projects, and innovative actions that provide smart interactive ICT systems, which implement the concept of eHealth to maintain the cognitive status of users while providing novel ways of rehabilitation.

### Individual Assessment of Stroke Patients

Some of the most important aspects taking into account the traditional techniques for assessment of stroke patients and cognitive rehabilitation are related to the following:

- The analysis of the manipulation of single tools, which demonstrates the existence of performance deficits even in the execution of simple activities
- The importance of the use of kinematics as a quantitative approach to analyze the performance of an action with tool use
- The fact that many goal-direct movements (ie, pointing) are impaired even in the hand ipsilateral to the lesion

#### Analysis of Tool Use

Testing the use of actual tools with real target objects has been mandatory to analyze the ability of stroke patients in interacting with objects in their daily living. Table 3 shows a summary of the main publications and tests carried out in this matter.

#### Analysis of Kinematics When Manipulating Tools

The temporal and spatial features of the movements are subaspects in some of the scoring methods, but the assessment is very rough. Nevertheless, there are other tests focused on these specific aspects, which monitored user movements in 3D space by using the adequate methodology for analyzing kinematics (Table 4).

#### Execution of Goal-Directed Movements

Bearing in mind that the studies that consider the recording of user movements during tool use are relatively rare in patients who suffer from left brain damage (LBD) or right brain damage (RBD), there is a great number of research on the kinematics of more basic goal-directed movements such as pointing, aiming to targets, or grasping neutral objects.

As indicated in Table 5, these tests noted specific deficits in patients with LBD versus patients with RBD, more dynamic aspects of movement following damage to the motor-dominant left hemisphere, and movement initiation and movement accuracy following damage to the right hemisphere.

**Table 3:** Summary of reports on the assessment of performance deficits when using objects in stroke patients. LBD: left brain damage.

Reference	Objects used	Result	Type of scoring
Liepmann [8]	Comb, brush, hammer	Errors in 25% of “dyspraxics” (N=42)	Right or wrong
De Renzi and Luchelli [36]	Common-use objects	All of them made errors	Major or minor or no error
McDonald et al [37]	Cup, key, fan, scissors	17 LBD patients: no differentiation from other task modes	Right or wrong
Buxbaum et al [38]	Common-use objects	Single case: fewer errors during use	Grasp, trajectory, amplitude, and timing
Westwood et al [39]	Hammer, saw, spectacles	Object use deficit: 37 LBD patients (43%); 50 right brain damage patients (18%)	Performance accuracy from composite scores
Goldenberg et al [40]	Glass, apple, electric bulb, squeezer	10 LBD patients: more errors in the use of actual tools	The presence of feature for grasping and movement
Randerath et al [41]	Hammer, ladle	25 LBD patients: errors in almost all conditions	The presence of features: grasp, movement execution, direction, space

**Table 4:** Summary of reports about the analysis of 3D movement kinematics when manipulating objects. LBD: left brain damage ; RBD: right brain damage.

Reference	Task	Result
Clark et al [42]	Slicing bread	3 LBD patients: imprecise plane of motion and trajectory shape
Poizner et al [43]	Slicing bread	3 LBD patients: impaired joint coordination
Laimgruber, et al [44]	Grasping a glass	19 LBD patients + 10 RBD patients: prolonged adjustment phase
		RBD: slowed velocity
Hermsdörfer et al [45]	Sawing	9 LBD patients: velocity deficits
Hermsdörfer et al [46]	Hammering	23 LBD patients: prolonged reaction time, slowed velocity.
		10 RBD patients: prolonged reaction time
Hermsdörferet al [47]	Scooping	23 LBD patients: reduced amplitude, reduced hand roll
		9 RBD patients: no deficits

**Table 5:** Summary of studies on deficits during goal-directed movements with the ipsilesional hand. LBD: left brain damage; RBD: right brain damage.

Reference	Task	Result in patients
Hermsdörferet al [48]	Grasping	LBD: acceleration deficits
		RBD: adjustment deficits
Hermsdörfer et al [49]	Grasping and placing	LBD: slowed movement, awkward hand rotation
		RBD: prolonged reaction time, slowed movement, hand placement errors
Schaeferet al [50]	Shoulder or elbow aiming	LBD: reduced acceleration amplitude
		RBD: reduced acceleration duration
Tretriluxana et al [51]	Grasping	LBD: deficient scaling of grasp preshaping
		RBD: weak transport-grasp coordination
Schaeferet al [52]	Shoulder or elbow aiming	LBD: impaired multijoint coordination
		RBD: decreased final accuracy
Schaefer et al [53]	Visuomotor adaptation	LBD: initial direction adaptation impaired
		RBD: final adjustment impaired
Haaland et al [54]	Elbow aiming movements	LBD + paresis: reduced amplitude modulation
Mutha et al [55]	Visuomotor adaptation	LBD + apraxia: impaired
Mutha et al [56]	Visuomotor adaptation	LBD parietal damage: impaired

### Traditional Methodologies to Support Cognitive Rehabilitation

The implementation of intelligent environments to provide a rehabilitation platform is something relatively new, which many researchers are focusing their efforts on. In fact, later in the study, the most important work exploring this topic will be presented.

Once a general view of how to assess the level of severity of stroke patients, as well as to screen them, has been presented, the main traditional techniques to provide cognitive rehabilitation in their daily living are summarized. As derived from the reading of this subsection, classical techniques are not especially based on the use of smart technology, which means high workload for therapists and clinicians along with long and frequent patient visits to the hospitals or rehabilitation centers.

Table 6 shows a summary of different approaches that provide cognitive rehabilitation once the corresponding stroke patients have been screened. The majority of the methods presented try to improve ADL performance and to increase the independence of the patients. For example, the execution of a task can be improved by the personalized feedback prompted to the patients [26] and by breaking the task down into basic actions [57].

**Table 6:** Traditional approaches for cognitive rehabilitation after stroke. ADL: activities of daily living.

Approach	Description	Result
Strategy training approach [58]	Internal and external compensatory strategies	Strategy training groups improve patients’ dexterity
Errorless learning [11]	Manipulation of limbs during ADL	Significant improvement on trained activities
	Simultaneous performance of ADL with therapist or examiner	
Variety of approaches [57]	Pictorial representation of the goals and subgoals, written commands	No significant effects on trained tasks
Verbalization strategy [26]	Patient taught a poem based on the steps of making a cup of tea	Weak training effects across sessions and no transfer to untrained tasks or objects
Error monitoring and detection; task training action intervention [59]	Pictorial descriptions of objects	Better performance on the Naturalistic Action Test
	Video presentation of the task, from a patient’s perspective	

### Information and Communication Technology–Based Personalized, Long-Term, and Continuous Cognitive Rehabilitation Systems

Considering the limitations presented in traditional techniques, these methodologies do not offer many benefits during the rehabilitation stage. For that reason, efforts have been taken in the previous years to develop systems that monitor the performance of a task and provide feedback, making it familiar, personalized, and attractive for the user. Successful execution of rehabilitation tasks would increase and the use of a smart environment (even at patients’ house) would improve independence in daily living and would alleviate occupational therapists' workload.

This subsection aims at describing the main research approaches and projects in the last 15 years related to providing smart platforms or environments that support cognitive rehabilitation and even maintain the cognitive status of the elderly, empowering their active aging.

#### Approaches Proposed in the Literature

Table 7 shows a description of 6 main eHealth-based approaches focused on cognitive rehabilitation. They have been classified based on relevant research publications.

#### European Projects

There are 9 main European research projects focused on the development of prototypes based on new technologies for providing advanced cognitive rehabilitation, excluding those centered in the rehabilitation of the movement of body segments. These are explained in detail in Table 8.

**Table 7:** Summary of published approaches focused on information and communication technology–based solutions for cognitive rehabilitation.

Approach	Description	Result	Main feature
Remote acquisition of neuropsychological data [60]	An architecture that allows collaborative video conferencing and continuous virtual interaction with patient	Data are successfully obtained from patients who are not familiar with technology	Home based Accessible
Living Labs [61]	Interaction with real world is monitored by health care sensing	Living Labs improves independence and quality of life	Home based Monitoring of the execution of complex daily activities Accessible
Virtual reality [62]	A virtual reality–based prototype to improve coordination skills of stroke patients	A technology-assisted solution that improves endurance abilities	Home based Automatic error detection
Noninvasive, open, and distributed architectures (RehabNet) [63]	A system based on neuroscience that provides an interactive interface for stroke rehabilitation	Patients with high spasticity had better control by using a regular glove	Home based Automatic error detection
Brain-computer interfaces [64]	Communication tool to support neuronal plasticity by activating language circuits	Aphasia patients initially had problems to use the paradigm of the visual speller	Automatic error detection
Tele-stroke [65-68]	Wireless telemedicine and mobile apps: teleradiology [66]	This improves the efficiency of the usage of resources as well as the interaction with patients	Home based Monitoring of the execution of complex daily activities Accessible
Robots [69]	A socially assistive robotic platform to propose and adopt new plans to new situations in real time	It maintains verbal and nonverbal communication with users	Home based
Dashboard design [70]	Use of an interactive dashboard platform to assess upper limb movements in daily living	It improves the acquisition of users’ data, engagement of patients, and coordination between clinicians	Home based Monitoring of the execution of complex daily activities Accessible

**Table 8:** European research projects focused on information and communication technology–based cognitive rehabilitation.

Project	Result	Main feature
MIMICS: Multimodal Immersive Motion Rehabilitation with Interactive Cognitive Systems [71]	Immersive multimodal virtual environments for sensory motor rehabilitation	Monitoring of the execution of complex daily activities Accessible
COACH: Cognitive Orthosis for Assisting with Activities in the Home [72]	Smart platform to supervise elderly Alzheimer’s patients	Home based Automatic error detection Accessible
GUIDE, Technology for Independent Living [73]	Prototype to assist stroke patients in the learning and execution of laundry and dressing tasks	Home based Monitoring of the execution of complex daily activities
DEM@CARE: Dementia Ambient Care: Multi-Sensing Monitoring for Intelligent Remote Management and Decision Support [74] CONTRAST: Remote Control Cognitive Training [75]	Adaptive human-computer interaction for neuro feedback training in dementia patients	Home based Accessible
COGWATCH: Cognitive Rehabilitation of Apraxia and Action Disorganisation Syndrome [76]	Information and communication technology (ICT) prototype for the cognitive rehabilitation of patients with apraxia and action disorganization syndrome in real time [77]	Home based Automatic error detection Monitoring of the execution of complex daily activities Accessible
VR STROKE REHAB: Virtual Reality Intervention for Stroke Rehabilitation [78]	Use of virtual reality to encourage chronic stroke patients	Home based
HOMER: Development of Home Rehabilitation System [79]	Open-access platform for cognitive rehabilitation, which integrates virtual reality and ICT commercial systems	Home based Automatic error detection Monitoring of the execution of complex daily activities Accessible
SWORD: Advanced Analytics Platform for Stroke Patients Rehabilitation [80]	Integration of current technologies into novel neuroscience-driven therapeutic methods	Home based Monitoring of the execution of complex daily activities Accessible
ACTIVE HANDS [81]	Multimodal platform at home to provide user feedback in daily tasks	Home based Monitoring of the execution of complex daily activities Automatic error detection Accessible

#### Other Coaching Platforms and Large-Scale Innovative Actions

Elderly people wish to live at home and independently as long as possible. Different multidisciplinary research groups are nowadays working on achieving the “smart house of the future” to suit the needs of people. For example, the Orcatech project is focused on carrying out a pilot study to determine adherence to treatment of an internet-based platform that provides mindfulness meditation [82]. The platform uses what is called a life laboratory as a resource to explore technologies that support independent living, to assess new behavioral markers, and to evaluate approaches for assessing neurological and other relevant health changes, all in the participant’s home.

The Gloucester “Smart House” project for people with dementia supports this population in their ADL and focused on the cognitive aspects [83]. The smart house has all the necessary equipment found in a conventional house but with different sensors and control panels connected to remind and help in different daily tasks and security aspects: leisure activities, bathroom flooding, cooker monitor and fire usage, falling, nighttime wandering, taking medication, and forgetting keys.

Meanwhile, “UTA’s MavHome Smart House” project physically assists the elderly and individuals with cognitive disabilities by providing home capabilities that will monitor health trends and assist in the inhabitant's day-to-day activities in their own homes [84].

Finally, City4Age aims at enabling “Ambient Assisted Cities” by defining elderly-friendly city services for the active and healthy aging. The core idea is to demonstrate that “smart cities” can play a pivotal role in the early detection of mild cognitive impairment (MCI) and frailty risks and subsequent interventions by collecting data about individual behaviors in an unobtrusive way [85]. Data collected are used twofold: first, to cluster population segments that show MCI and frailty evidence; second, to pay attention and monitor closely the population at risk already identified. When a change of behavior that may lead to a risk of MCI or frailty is detected, an intervention is provided to the user with the aim of persuading the user to reverse these changes to a positive behavior and thus reduce risks.

The City4Age project lead by Politecnico di Milano makes use of current city services (open data) and collected large amount of data about citizens, such as usage of public transports, services, and shopping, to develop useful interventions. Interventions could include ecological momentary interventions to improve the quality of life in daily living, dynamic interventions providing positive patterns of behavior change, and just-in-time interventions to assist individuals in case a higher priority intervention is required.

## Discussion

### Principal Findings

The material presented in this study addressed different topics relevant for the cognitive rehabilitation of stroke patients and the importance of ICT technologies as well as the implementation of eHealth and tele-rehabilitation concepts for the evolution of classical methodologies. Moreover, the concept and consequences of the most common syndromes after stroke were described along with an introduction.

From the review of the literature and regarding the assessment of the performance of stroke patients during the use of single tools for their individual screening, 7 main traditional relevant works have been presented as well as 6 examples of methods that include analysis of kinematics, and 9 focused on the performance of goal-oriented tasks.

First of all, the analysis of single tool use has emphasized the role of the left hemisphere of the brain in this type of tasks (eg, use of comb, hammer, glass) and also showed that actual task performance is generally less compromised than out-of-context performance. However, most of the tests demonstrated that a high number of patients present deficits even in the performance of seemingly simple activities. Second, the analysis of kinematics has also been considered as useful in the analysis of actual tool use. As derived from the tables presented, this kind of analysis is more sensitive than scoring systems. Finally, the results of some studies noted that the processing and interpretation of the data acquired from patients’ behavior for the recognition of movements, which makes possible the determination of the success of an action, can be affected by more elementary deficits of the patients during the performance of goal-directed movements.

Meanwhile, the classical methods to provide cognitive rehabilitation once the screening of the corresponding stroke patients is done have been classified in 5 main techniques. On the basis of the analysis of the results, there is evidence that new rehabilitation technologies are needed. Among the main reasons why new ICT systems are necessary, the following ones can be derived:

- There are a high number of stroke survivors who need long-term cognitive rehabilitation.
- Consequences and traditional rehabilitation techniques of apraxia and action disorganization syndrome reduce independence.
- The economic costs of health care are significant when dealing with rehabilitation after stroke.
- Stroke patients show improvements very slowly by using current hospital-based rehabilitation methods.
- One of the main consequences of the inadequacy of current techniques is the fact that many patients present lifelong disabilities and suffer from social exclusion.

These statements are directly related to the questions proposed and indicated in the methodology. For that reason, the review has covered the main current instances of ICT–based cognitive rehabilitation systems or approaches. The study has been completed with 8 different technology-based approaches from scientific-technical literature; 9 European projects from past Framework Programs to the current Horizon 2020; and other actions such as the use of virtual coaching, 3 examples of smart houses, and the large-scale initiative City4Age. This compilation of projects has been limited to the last 15 years approximately.

### Limitations of Smart Technologies for Daily Living

Although nowadays ICT–based platforms are the main interest of research for many professionals and institutions to provide cognitive rehabilitation after stroke, current research initiatives focused on providing such smart systems for coaching and maintenance of cognitive well-being should be well aware of some barriers and take them into account when designing and developing the smart environments.

In terms of user acceptance, older adults are mainly using the internet and mobile phones to keep in contact with family and friends. Although the proportion of older adults using these technologies is less than in any other age, the numbers are steadily increasing [86,87]. There are also some studies focused especially on the acceptance of users in using their mobile phones to monitor health status [88,89]. Nonetheless, assistive technologies especially for cognitive rehabilitation still need to overcome significant barriers to be adopted by older people including privacy, functionality, suitability for daily use, perceived need and usefulness, costs, accessibility, fear of dependence, lack of training, and stigmatization by using technologies specifically targeting older users (“gerontechnology”) [90,91].

To deal with this issue, focus groups should be involved in the user requirements and throughout the development of the prototypes. In addition, user groups should be recruited to validate the components developed in different cycles. A user-centered approach would ensure that the usability and perception of the usefulness of the systems are maximized. Moreover, cost barriers must also be addressed by aiming to use affordable, off-the-shelf technologies.

**Figure 1:**
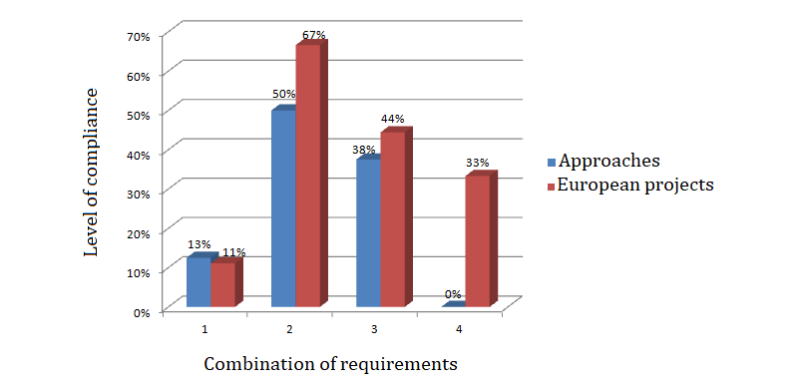
Level of compliance of the approaches and European projects presented based on the requirements to fulfill.

### Conclusions

Stroke has significant negative effects on the socioeconomics of countries. Considering the health budget allocated for stroke in Europe and with the goal of improving the efficacy of current rehabilitation techniques and the personal life of stroke patients, the future implementation of systems that are able to provide cognitive rehabilitation at home will have a positive impact on daily living.

Increasing the independence of stroke patients can improve their personal lives because self-confidence of patients will improve as well as their socialization with other family members and friends. It is quite important to ensure proper emotional status in patients which will make the acceptance of rehabilitation easier. Moreover, increasing the personal independence of stroke patients will have a direct implication for health care services.

From the review of current state-of-the-art ICT–based approaches for cognitive self-rehabilitation and tele-rehabilitation, the most relevant ones have been presented in this document. Figure 1 shows the level of compliance of the approaches and European projects described with the requirements mentioned in the methodology considering the following assumptions:

1. Only home-based performance
2. Home-based performance + accessibility
3. Home-based performance + accessibility + support in the execution of complex daily living tasks
4. Home-based performance + accessibility + support in the execution of complex daily living tasks + automatic error detection

By analyzing the data, it can be derived that none of the approaches found in the literature provide all the 4 features at the same time, and only 50% of them are accessible. Meanwhile, although 77% of the European projects presented are conscious of the importance of making new technologies accessible, bearing in mind the limitations of some patients, only 33% of them fulfill the 4 requisites considered as essential.

For this reason, current and future large-scale initiatives focused on smart environments should try to present all these features to users. The design and use of personalized and eHealth rehabilitation systems, which could be used for the assessment of a wide range of neurological disorders including those syndromes not presented in this study, will reduce hospitalization rates as well as the frequency of home visits by health professionals, which means a reduction in costs for the national health care services.

## Abbreviations

ADL: activities of daily living
ADS: action disorganization syndrome
CORDIS: Community Research and Development Information Service
ICT: information and communication technology
LBD: left brain damage
MCI: mild cognitive impairment
RBD: right brain damage
